# Added value of contrast-enhanced ultrasound (CEUS) in the diagnosis of primary retroperitoneal serous adenocarcinoma: a case report

**DOI:** 10.1186/s12880-021-00613-4

**Published:** 2021-05-12

**Authors:** Lin-Yu Zhou, Xiao-Dan Zhu, Jian Jiang, Yan-Feng Bai, Tian-An Jiang

**Affiliations:** 1 Department of Ultrasound Medicine, The First Affiliated Hospital, Zhejiang University School of Medicine, Hangzhou, Zhejiang Province People’s Republic of China; 2 Department of Pathology, The First Affiliated Hospital, College of Medicine, Zhejiang University, No. 79 Qingchun Road, Hangzhou, 310003 Zhejiang Province People’s Republic of China; 3 Zhejiang Provincial Key Laboratory of Pulsed Electric Field Technology for Medical Transformation, Hangzhou, Zhejiang Province People’s Republic of China

**Keywords:** Retroperitoneal space mass, Primary retroperitoneal serous adenocarcinoma, Contrast-enhanced ultrasound, Ultrasound

## Abstract

**Background:**

Primary retroperitoneal serous adenocarcinoma (PRSA) is a rare malignant disease. Given the rarity of the disease, the imaging features of PRSA are unclear. Contrast-enhanced ultrasound (CEUS) also plays an important role in the evaluation of the differential diagnosis of retroperitoneal lesions.

**Case presentation:**

We report the case of a 62-year-old woman of with increased CA125 levels for 1 year who was referred to our hospital. After conducting contrast-enhanced computed tomography and magnetic resonance imaging, the mass was misdiagnosed as a chocolate cyst. After transvaginal ultrasound (TUS) combined with CEUS, cystadenocarcinoma was considered as the initial diagnosis. Pathology results confirmed PRSA as the final diagnosis.

**Conclusions:**

CEUS features of PRSA are reported for the first time based on this case, potentially aiding in the differential diagnosis of this rare entity before surgery.

## Background

Pelvic tumours are commonly identified as primary gynaecological tumours or metastatic tumours. However, most pelvic masses cannot be detected early due to their location. The tumour often grows to a large size before symptoms appear and is often found by accident or upon examination. Given the rarity and unknown biological behaviour of primary retroperitoneal serous adenocarcinoma (PRSA), its imaging features are unclear. PRSA was first reported by Ulbright et al. in [1], and since then, fewer than 15 cases have been reported in the literature.

In this manuscript, we describe the ultrasound and contrast-enhanced ultrasound (CEUS) imaging features in patients with PRSA and review the relevant literature. To the best of our knowledge, this is the first report on the CEUS findings of PRSA.

## Case presentation

A 62-year-old woman was found to have an elevated serum CA125 level of 50 U/ml (normal < 35 U/ml) at a local hospital in November 2018. Her serum CEA and CA19-9 levels were within normal limits, and no obvious abnormalities were found on transvaginal ultrasound (TUS). In November 2019, at the same hospital, her CA125 level had increased to 75 U/ml. TUS performed at the local hospital revealed a large pelvic mass.

She was then referred to our hospital for further diagnosis and treatment. Laboratory test results, including assessment of tumour markers, routine reproductive hormone examination and routine blood tests, were within the standard levels. The patient was asymptomatic, and a physical examination revealed no abdominal mass. The patient’s family history indicated that the patient’s mother died from ovarian cancer.

**Fig. 1:**
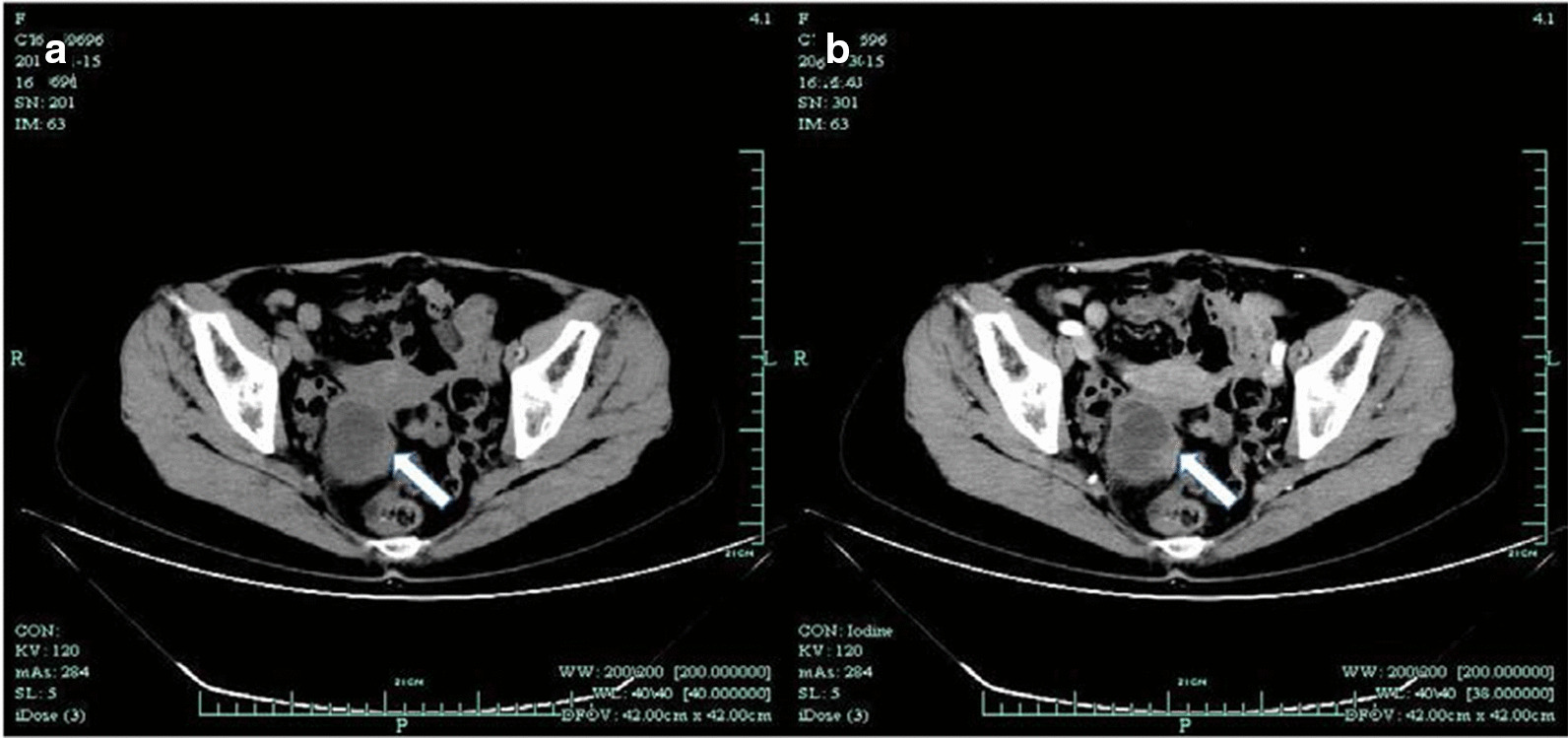
**a** Axial CT image showed s a well-defined heterogeneous mass with central hypodense areas in the right retroperitoneal space (arrow). **b** Contrast-enhanced CT scan showed hyperattenuating of papillary excrescence of the mass (arrow)

**Fig. 2:**
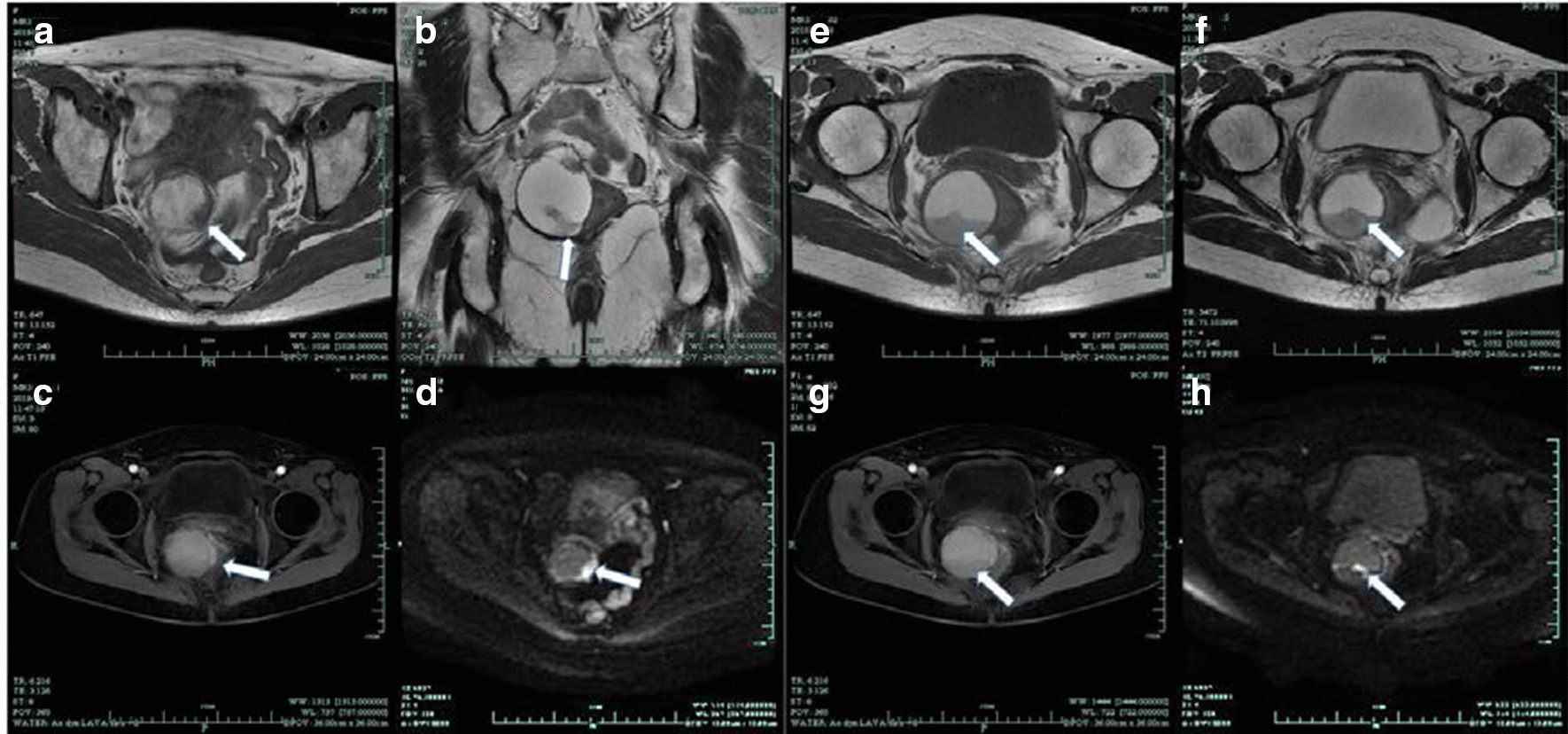
The magnetic resonance imaging findings of the mass. **a**–**d** The MRI imaging findings of the mural nodule of the tumour. **e**–**h** The MRI imaging findings of the septal nodule of the tumour. **a** and **e** T1-weighted MR images; **b** and **f** T2-weighted MR images; **c** and **g** on Gadolinium-enhanced T1-weighted MR images at arterial phases, the mural and septal nodule of the tumour showed heterogeneous enhancement. **d** and **h** The papillary excrescences of the tumour (arrow) showed high signal intensity on diffusion weighted imaging. The pathological finding was high-grade serous adenocarcinoma

**Fig. 3:**
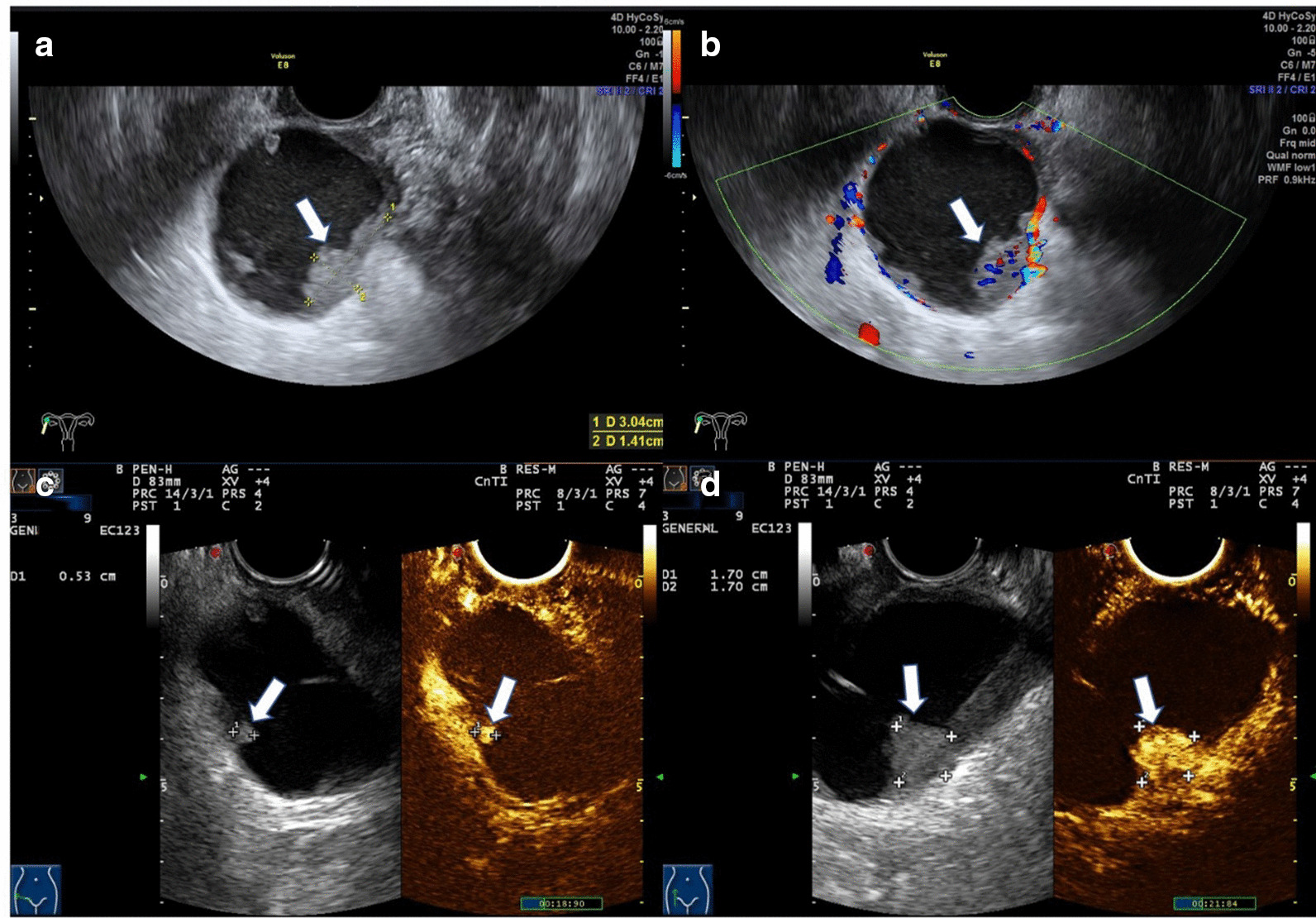
Ultrasound appearance of the PRSA. **a** Conventional ultrasound shows an unilocular cystic lesion with papillary excrescences measured 3.0 * 1.4 cm (arrow). **b** Color Doppler image shows the solid papillary projection contains internal flow (arrow). **c**, **d** Hyper-enhancement of cystic wall, intracystic septations, and intracystic solid components is found in the arterial phase on CEUS (arrow)

Abdominal computed tomography (CT) revealed a right adnexal well-circumscribed hypodense mass (Fig. 1). On a contrast-enhanced CT scan, slight enhancement of the mass was noted and a fluid–fluid level sign was observed. Routine and contrast-enhanced MRI demonstrated well-defined lobulated cystic mass in the right pelvic cavity that was approximately 4.9 × 4.8 cm in size (Fig. 2). The mass was of mixed signal on T2-weighted imaging (T2WI) and T1-weighted imaging (T1WI). Nodular hypointensity on both T1WI and T2WI was observed within the lesion. Diffusion weighted imaging (DWI) showed severe hyperintensity. A fluid–fluid level sign was also observed in the cystic mass. The patient was suspected of having a chocolate cyst according to the clinical CT and MRI results. Routine preoperational TUS showed a right adnexal mass with a size of approximately 5.6 × 4.4 × 5.5 cm. Conventional ultrasound showed a well-defined cystic mass with mural and septal nodules. The largest size of internal papillary excrescences was 3.0 × 1.4 × 1.7 cm. Doppler flow could be detected in the internal septation, cystic wall and papillary excrescences. The resistive index of the cyst wall was 0.9. The right ovary was visible. There was no ascites. Considering the appearance and features on TUS, a primary diagnosis of malignant neoplasm was made. To further clarify the nature of the lesion, the patient underwent a CEUS examination, during which 2.4 ml of ultrasound contrast agent (SonoVue; Bracco SpA, Milan, Italy) was injected from the peripheral vein by bolus injection. Hyperenhancement of cystic wall, septations, and papillary excrescences were observed in the arterial phase on CEUS. The mass becomes hypo-enhancement in the late phase. The mural-nodule-like solid component measured approximately 1.7 × 1.7 cm and the smaller one 0.5 × 0.5 cm (Fig. 3). Based on the contrast ultrasound results, the diagnosis of cystadenocarcinoma was first considered.

**Fig. 4:**
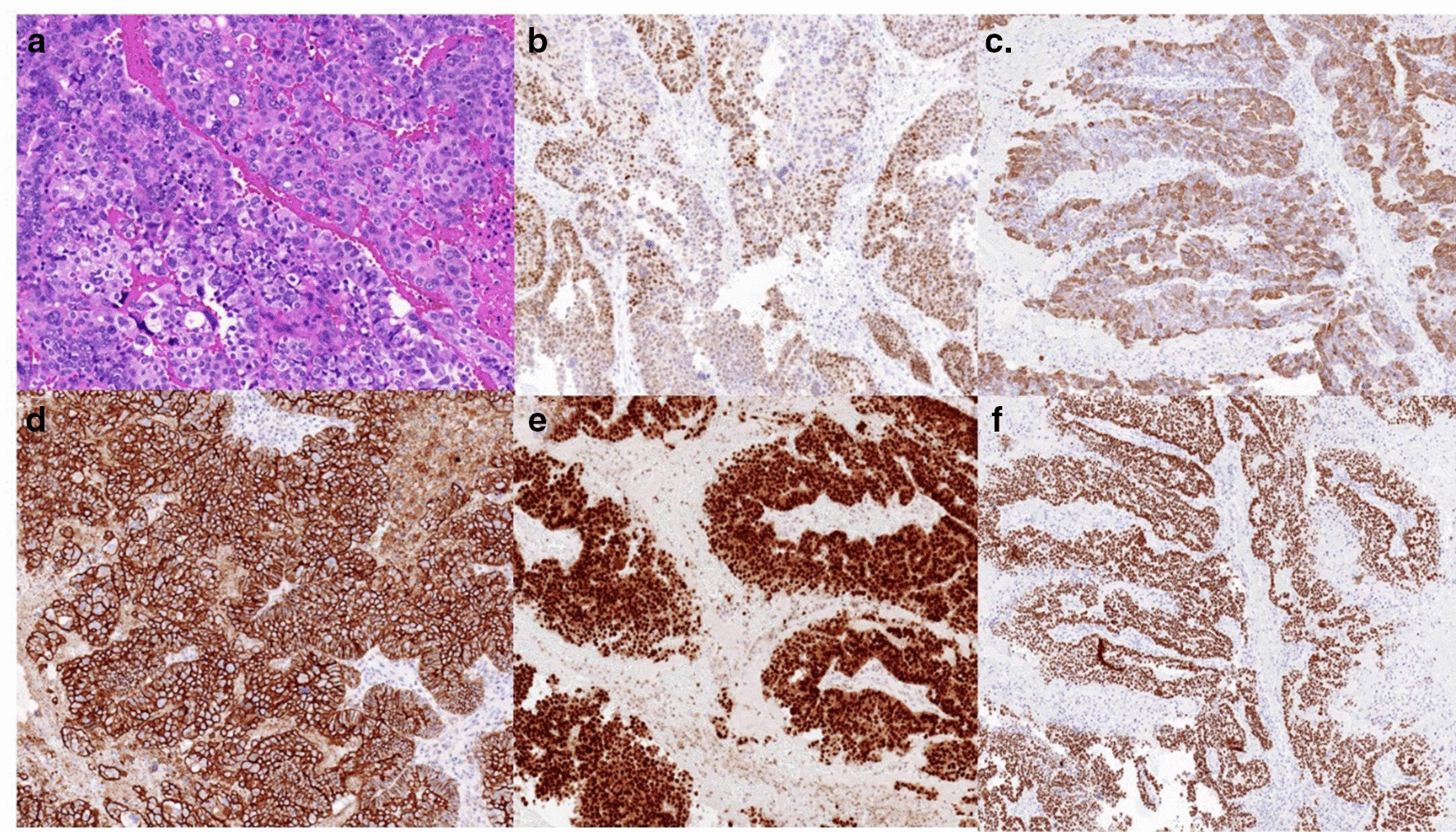
Histopathological examinations of the tumour. **a** Histopathological examination with haematoxylin and eosin staining of the tumour showed that tumour cells were arranged in a nested papillary structure (×400). **b**–**f** The tumour cells were positive for oestrogen receptor (**b**; ×200), CK7 (**c**; ×200), CA125 (**d**; ×200), PAX8 (**d**; ×200), and WT1 (**f**; ×200)

Surgical resection of the retroperitoneal mass was performed. During the operation, a mass approximately 5 cm in diameter was found in the Douglas cul-de-sac. It was soft and had no obvious adhesion to surrounding tissues. The tumour was cystic and solid, the cyst wall was thick, and the cyst contained solid tissue. Both ovaries and fallopian tubes are normal. Frozen biopsy showed (pelvic) adenocarcinoma, mainly with papillary growth. Da Vinci robot-assisted laparoscopic pelvic tumour resection was conducted. Expert pathologists with more than 10 years of experience determined the pathologic results. Grossly, the tumour was multi-cystic, well-encapsulated and filled with serous fluid. The inner surface contained several papillary nodules protruding into the cavity. Under microscopic examination, the tumour tissues were arranged in a papillary shape; a glandular tube, invasive growth, haemorrhage and necrosis were observed. The tumour cells were abnormally shaped, nuclear division was visible, and nucleoli were obvious. The immunohistochemistry results were as follows: CK20(−), CK7(−), p53(+), CA125(+), CDX2(−), PAX-8(+), ER (+), PR(−), WT(+), calretinin CR(−) and vimentin(+) (Fig. 4). The final pathological finding was high-grade serous adenocarcinoma. The patient’s serum CA125 levels decreased after the operation, but the level was slightly higher than the normal range (< 35 U/ml). The patient was treated with a combination of adjuvant carboplatin and paclitaxel (544 mg carboplatin and 268 mg paclitaxel intravenously every 3 weeks) at 3-week intervals for eight cycles. After two cycles of chemotherapy, her serum CA125 level decreased to a normal level. The patient has completed all chemotherapy cycles and is now in good condition. Her serum CA125 level has remained within the normal range as of publication of this report. Written consent was obtained from the patient for publication of the case.

## Discussion and conclusions

Primary retroperitoneal tumours are uncommon and account for 0.2–0.3% of all tumours. The histological types of posterior peritoneal masses are diverse. PRSA is an epithelial tumour that is histologically similarly to ovarian serous carcinoma. To date, there have been twelve reported cases of PRSA [1–12] (Table 1), all but one of which were in females. The histogenesis of PRSA tumours remains unknown. Several possibilities have been proposed, including metaplastic coelomic epithelium, extraovarian endometriosis [7], heterotopic ovarian tissues [2, 5, 7, 13] and cystic endosalpingiosis [6, 14]. However, coelomic metaplasia is the most widely accepted tumour origin [2, 5–7, 10]. The secondary Müllerian duct hypothesis may also be applicable to the development of PRSA. However, in most patients, including the patient in the present study, no ovarian stroma was found around the tumour, which may be contrary to the hypothesis of heterotopic ovarian tissue. Most cases of high-grade ovarian serous carcinoma are thought to be caused by the fallopian tube. Given the genetic relationship between retroperitoneal high-grade serous carcinoma (HGSC) and serous tubal intraepithelial carcinoma (STIC) lesions, Suda et al. suggested that retroperitoneal HGSC might originate from STIC [12]. In our case and in some previous cases, bilateral salpingo-oophorectomy was performed, but there was no sign of STIC coexistence. Therefore, this view requires further confirmation.

**Table 1:** Retroperitoneal serous adenocarcinoma cases reported since 1983

	Author	Year	Age/sex	Initial presentation	Site	Size (mm)	Grouth pattern	Elevated tumor markers	Operation	Chemotheraphy	Outcomes
1	Ulbright	1983	11/F	Weight gain	Encased the right common iliac artery	L80 × 130 × 110	Solid	None	Partial resection	Chemotheraphy	NED 10 months
2	Caruncho	1993	12/F	Abdominal pain	Adjacent to the kidney	60	Solid and cystic	CEA	Partial resection	None	Not mentioned
3	Kurosaki	1998	38/F	Abdominal distention	Adherent to the kidney	90 × 60 × 50	Cystic	CEA	Complete resection with partial nephrectomy	None	NED 24 months
4	Fujiwara	1999	54/F	Abdominal distention	Adjacent to aorta	Not mentioned	Invasive	CA125	None	Chemotheraphy	DOD 24 months
5	Kaku	2004	44/F	None	Adjacent to the left psoas major muscle	60 × 35 × 30	Cystic	CA125, CA19-9	Complete resection with a partial resection of the psoas muscle	None	AWD 23 months
6	Iura	2009	66/F	Abdominal pain	Adherent to the ileocecum	200 × 95 × 85	Solid	CA125, CA19-10	Complete resection with a partial resection of the psoas muscle	Adjuvant chemotheraphy	AWD 32 months
7	Arichi	2011	75/F	None	Adherent to the diaphragm	38 × 47 × 50	Cystic	CA125	Complete resection with a partial resection of the diaphragm	Adjuvant chemotheraphy	NED 6 months
8	Zhang	2017	58/F	Alternate stool abnormality	Adjacent to the douglas pouch	43 × 33 × 26	Solid	CA125	Complete resection with a partial resection of onentum	Adjuvant chemotheraphy	AWD 30 months
9	Kohada	2017	42/F	Left back pain	Adjacent to the lower pole of the left kidney	55 × 62 × 55	Cystic	CA19-9	Complete resection with a partial resection of peritoneum	Adjuvant chemotheraphy	NED 3 months
10	Nakatake	2018	74/F	A tumor of the liver	Right retroperitoneal cavity and liver	15 and 20	No description	None	Complete resection with partial hepatecomy	None	NED 12 months
11	Suda	2019	58/F	Persistent defecation disorder and vomiting	In the mesorectum	80 × 55 × 35	Solid and cystic	CA125	Complete resection with a partial resection of the rectum	Adjuvant chemotheraphy	NED 20 months
12	Chae	2019	71/M	Right-side back pain and numbness	In the right retropertioneum	91 × 53 × 140	Solid and cystic	None	None	Adjuvant chemotheraphy and external radiotherapy	AWD 15 months

*CEA* carcinoembryonic antigen, *CA125* cancer antigen125, *CA19-9* cancer antigen19-9, *AWD* alive with disease, *NED* no evidence of disease, *DOD* died of disease

Primary retroperitoneal masses include various neoplastic and non-neoplastic entities that appear in the retroperitoneum but are not derived from any retroperitoneal organs and are typically uncommon. After we have confirmed the location of the tumour, we need to rule out the possibility that it arises from a retroperitoneal organ. Then according to the imaging performance, the mass can be classified as solid or cystic [15]. In this case, the retroperitoneal mass was mainly cystic. In the differential imaging diagnosis, other cystic or pseudocystic retroperitoneal tumours must also be considered [16, 17], such as cystic lymphangioma, leiomyoma, teratoma and neurogenic tumours. A clinical history may facilitate the diagnosis of non-neoplastic lesions, such as pancreatic pseudocyst, lymphocele, urinoma and hematoma. Patients with lymphatic cysts typically have a recent history of surgical lymphadenectomy. Patients with pancreatic pseudocysts often have a history of pancreatitis and high levels of amylase or lipase. Patients with urinoma or hematoma generally have history of trauma. Our patient had no previous history, so these diagnoses can be excluded. Neoplastic masses include cystic lymphangioma, cystic mesothelioma, epidermoid cyst, cystic teratoma, and ovarian cystadenoma.

In addition to clinical features, the specific characteristics of various retroperitoneal tumours, such as spreading pattern, tumour composition, and vascularity, all contribute to the differential diagnosis [18]. Cystic lymphangioma may cross from one retroperitoneal compartment to an adjacent compartment. Cystic lymphangioma, cystic mesothelioma and epidermoid cysts typically present as thin-walled, unilocular or multi-locular cysts [19]. The presence of calcification in the cyst wall highly suggests the possibility of cystic teratoma. Vascularity is another important feature of retroperitoneal mass. The blood perfusion of the cyst wall and intralesional solid component can be clearly displayed in CEUS and provides powerful clues, which can help narrow the scope of the differential diagnosis.

The overall appearance of PRSA was cystic in five cases, and a combination of solid components and cystic lesions was observed in the final case. These findings indicate that PRSA tends to have a cystic distribution and local growth in the retroperitoneal area.

In our case, the patient underwent a CT scan, MRI, TUS and CEUS to identify the nature of the mass. After both CT and MRI, the mass was misdiagnosed as a chocolate cyst. Chocolate cysts are also called ovarian endometriosis cysts. These masses are cystic lesions that are mainly thin-walled with smooth inner walls and no mutual or septal nodules. There will be no enhancement in any part of the lesion in contrast enhanced CT or MRI. And such patients usually present with dysmenorrhea. CEUS considered ovarian cystadenoma as the diagnosis. However, the diagnosis of ovarian cystadenoma needs to first confirm the existence of the affected ovary. Especially in postmenopausal women, the ovaries become smaller and there are no follicles, which is more difficult to find on TUS. In this case, the patient’s right ovary was normal, so we believed that the lesion can rule out the diagnosis of ovarian tumours.

Reviewing the CT and MRI examinations, we found that there were mural and septal nodules. By contrast, slight enhancement was detected on the cystic wall and mural or septal nodules on both CT and MRI (Figs. 1, 2). Diffusion weighted imaging (DWI) revealed severe hyperintensity of the nodules. This was consistent with the rapid and hyperenhancement of nodules in the arterial phase found by CEUS, which was conducive to the differential diagnosis of benign and malignant lesions. DWI also plays an important part in differentiating benign lesions from malignant masses. Mural or septal nodularity and their enhancement of the arterial phase after CEUS indicates the possibility of malignancy, but the patient's specific medical history and clinical manifestations must also be considered with this diagnosis.

There is no standard therapy for PRSA. Previous studies have shown eight cases of completely excised PRSA, including adjacent organs. Complete resection of the tumour is currently the main treatment for PRSA. Total hysterectomy and bilateral salpingo-oophorectomy are the surgical options that yield the most favourable outcomes. However, it is also important to consider the possibility of excessive infiltration in the case of PRSA. Kaku et al. [7] suggested that based on the histological similarity of retroperitoneal epithelial tumours and epithelial ovarian cancer, combined chemotherapy with docetaxel and carboplatin may represent a better choice for primary PRSA. The patient in our case report underwent total hysterectomy with bilateral salpingo-oophorectomy and omentectomy followed by chemotherapy. Because PRSA has the biological potential to produce epithelial ovarian cancer, adjuvant chemotherapy after surgical resection should be the top priority. Given the rarity of reported cases, the prognosis of PRSA remains unclear.

In conclusion, PRSA is a rare tumour. TUS and CEUS are helpful in the diagnosis of pelvic masses. CEUS features of PRSA are reported for the first time and might help in the differential diagnosis of this rare entity. Although an ideal PRSA treatment strategy has not been established, the combination of surgical resection and adjuvant chemotherapy may represent the best choice for patients with PRSA. Additional evidence from further reports is needed to clarify the imaging characteristics and optimal management of PRSA.

## Data Availability

Not applicable.

## Abbreviations

PRSA: Primary retroperitoneal serous adenocarcinoma
CEUS: Contrast-enhanced ultrasound
TUS: Transvaginal ultrasound
MRI: Magnetic resonance imaging
CT: Computed tomography
DWI: Diffusion-weighted imaging
CDFI: Colour Doppler flow imaging
RI: Resistance index
HGSC: High-grade serous carcinoma
STIC: Serous tubal intraepithelial carcinoma
